# Changes in Hoof Shape During a Seven-Week Period When Horses Were Shod Versus Barefoot

**DOI:** 10.3390/ani9121017

**Published:** 2019-11-22

**Authors:** Sara R. Malone, Helen M. S. Davies

**Affiliations:** 1 Department of Animal Sciences, Rutgers, The State University of New Jersey, New Jersey 08901-1281, NJ, USA; 2 Faculty of Veterinary and Agricultural Sciences, The University of Melbourne, Parkville, VIC 3010, Australia; h.davies@unimelb.edu.au

**Keywords:** horseshoe, farrier, hoof, horseshoe, soundness, biomechanics

## Abstract

**Simple Summary:**

This study explored changes in hoof shape related to the application of horseshoes. Past research has indicated that horse hooves change shape in response to gallop exercise, so this study sought to separate the changes due to exercise from changes due to wearing horseshoes. Amongst the most important findings were that proximal (coronary band) hoof circumference decreased more when horses were shod compared to barefoot, and hoof angle decreased when horses were shod but increased when horses were barefoot over a seven-week interval. A better understanding of the relationship between hoof shape and shoeing could help improve horse management practices.

**Abstract:**

This crossover study tested the hypothesis that hoof shape would differ after a seven-week period of horses (*n* = 11) wearing shoes versus barefoot. An ANOVA appropriate to a crossover design was used to assess the differences in the change in hoof shape over the seven-week period and significance was set at *p* < 0.05. Results are displayed as the mean difference for horses when shod versus barefoot ± the SEM for the left (L) and right (R) front hooves. Proximal hoof circumference (PHC) decreased when horses were shod and barefoot, but this decrease was greater when horses were shod (L −0.65 ± 0.16 cm; *p* = 0.0026; R −0.78 ± 0.13 cm; *p* = 0.0002). Hoof angle increased slightly when horses were barefoot and decreased when they were shod (L −1.70 ± 0.31°; *p* = 0.0004; R −1.84 ± 0.54°; *p* = 0.0079). Sole length decreased more when horses were barefoot, but this was only significant for the right fore (R 5.07 ± 1.06 mm; *p* = 0.0010). Solar circumference increased when horses were barefoot but decreased when shod (L −1.19 ± 0.41 cm; *p* = 0.0182; R −1.50 ± 0.31 cm; *p* = 0.0010). This is the first study to show a significantly lower PHC when horses were shod compared to barefoot. The study suggests that shod horses may benefit from a shorter shoeing interval to help mitigate the changes in hoof angle.

## 1. Introduction

There is little doubt amongst equine industry stakeholders that hoof health is integral to the soundness of a horse. The shape (or conformation) of the hoof may have serious implications for the soundness of the animal and hoof problems are a major cause of lameness in the horse [1,2,3]. Specific hoof conformations have been associated with an increase in the risk of injury. For example, racehorses with long toes and low heels are more likely to suffer musculoskeletal failure while racing [4] and horses that suffer catastrophic lower limb injuries during racing have a higher incidence of underrun heels [5]. It is unclear if hoof conformation is a direct (mechanical) cause of injury, or if injury occurs in association with other biomechanical, physiological or neurological complications. 

The domestic horse hoof is a continuously growing biological material that is frequently subjected to human intervention. Both hoof trimming and the individual farrier performing the trim have significant effects on the shape of the hoof capsule [6,7]. Although it is common for horses to wear shoes, there is a lack of basic scientific knowledge regarding the interaction between the horse hoof and the horseshoe. Research indicates that a horseshoe can have significant effects on the movement of the horse. The type of shoe a horse wears can increase impact accelerations and increase the risk of injury in racing thoroughbreds [8]. Even horses that are accustomed to a steel shoe demonstrate slight, but significant, differences in movement and loading of the distal limb after shoeing [9].

Previous research has found that during a period of race training the hoof decreased in hoof angle [10] and decreased in proximal hoof circumference (PHC) [11]. In the latter study by Decurnex et al., the racehorses were wearing shoes while they were in race training and barefoot if they were spelled for more than three weeks, therefore it is difficult to determine if the changes in PHC were due to the horseshoes or exercise training. Changes in hoof shape could be due to several factors, and research has shown that the season [12], distance and substrate of travel [13], and lameness [14] can all influence hoof shape. A study using unshod horses exercising on a treadmill reported that PHC did not change, but hoof angle increased over a four-week exercise training period [15]. This combined knowledge led to the current research design to investigate the effects of horseshoes on hoof shape. No previous work has focused on changes in hoof shape by comparing horses when shod versus unshod (barefoot). This study was designed to test the hypothesis that hoof shape would change when the horses were shod versus barefoot. 

## 2. Materials and Methods 

Eleven adult Quarter Horse mares (mean age: 13 years; range: 6 to 22 years) were used in this study. Horses underwent both treatments, shod and barefoot, in a crossover design. Horses were randomly assigned to their first treatment. Shod horses had the left and right front hooves shod, and the rear hooves were left bare. At the beginning of the study, in data collection 1, the horse’s feet were all trimmed by the same farrier and the first set of measurements were collected (Pre 1). Group A horses were shod (*n* = 5). Seven weeks later, during data collection 2, the shoes were carefully removed from shod horses, and all the horses had measurements taken (Post 1). The horse’s hooves were then trimmed and measurements were taken (Pre 2). Shoes were then applied to Group B (*n* = 6). After a second seven-week period the shoes were carefully removed and the last set of measurements (Post 2) were collected. 

**Figure 1:**
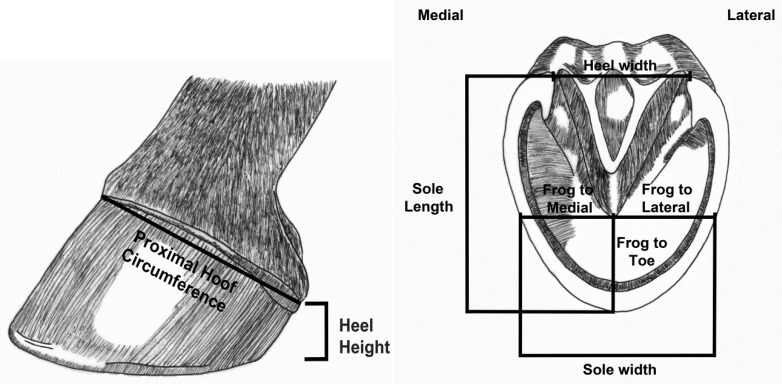
Measurements taken of the hoof.

**Figure 2:**
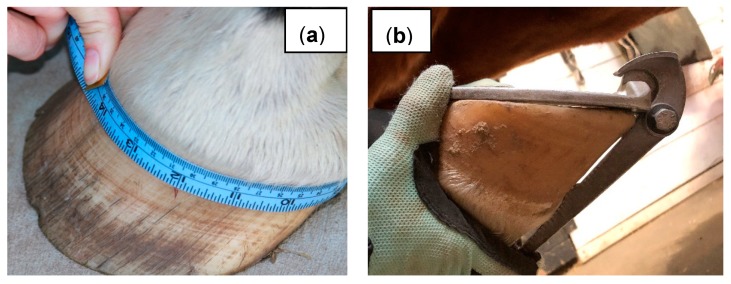
Measuring proximal hoof circumference (**a**) and hoof angle (**b**).

Duplicate measurements of both forelimbs were collected during each data collection, and the mean of the duplicates were used for analysis. The following measurements were taken directly from the horse hoof: PHC, hoof angle, frog to lateral wall, frog to medial wall, frog to toe, heel width, lateral heel height, medial heel height, sole length, and sole width (Figure 1). PHC was measured with a flexible seamstress tape using previously described methods [11], and hoof angle was measured using a Calvary hoof gauge (Figure 2). Other measurements were completed using plastic callipers. Tracings were made of the sole of the horses’ feet at each data collection. The horse stood on a piece of paper with a cardboard base and the hoof was traced. These tracings were later scanned and imported into ImageJ (Version 1.47b, National Institute of Health, Bethesda, MD, USA) software for the analysis of solar circumference and solar area. 

**Figure 3:**
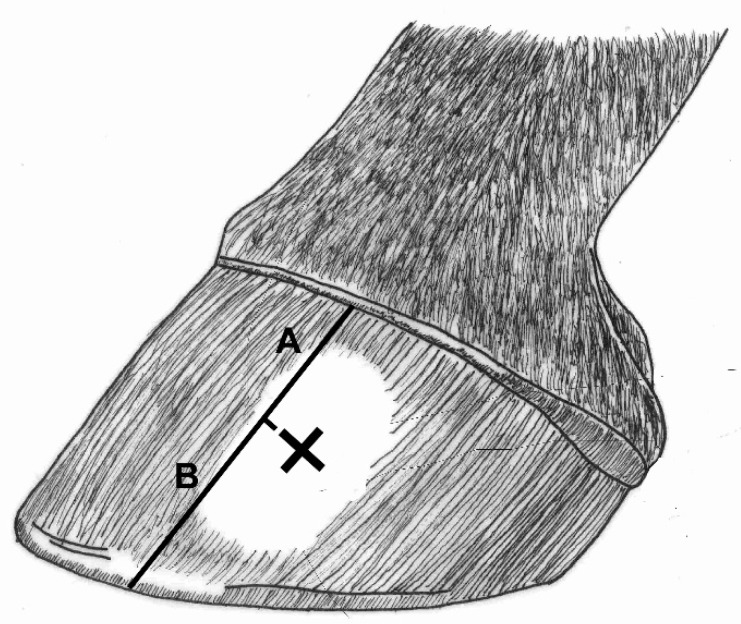
An example of a lateral cross mark burned into the horse’s hoof with a wood burning tool. The measurement of hoof growth (A) and hoof wear (B). The lines are offset for illustrative purposes only.

A standard horse weight tape was used to estimate body weight. To allow for an estimation of hoof growth and hoof wear, the hooves were hot branded at the beginning of each seven-week period with three cross marks: one on the lateral hoof wall (lateral cross mark), one centred on the dorsal hoof wall (dorsal cross mark), and one on the medial hoof wall (medial cross mark) (Figure 3). At each data collection, the distance from the coronary hairline to the center of the mark (hoof growth), and the distance from the mark to the ground (hoof wear), was recorded. This distance was measured with the grain of the hoof, as indicated in Figure 3.

Horses were unshod and maintained on a seven-week trimming interval (all trimming was performed by the same farrier) prior to the beginning of the study. The horses were housed at the same facility, managed under the same conditions, fed the same diet, and underwent no forced exercise during the trial (June to September). Horses were group-housed in paddocks with a mostly sand base. No horses received hoof supplements or hoof dressings. Three of the mares were lactating and pregnant; five mares were lactating and not pregnant; two mares were pregnant but not lactating; and one mare was neither pregnant nor lactating. Horses wore the same type of standard steel shoe in a suitable size. Hoof measurements were taken before the application of the shoe and after careful removal of the shoe by the qualified farrier. The farrier used the same standard trimming protocol that had previously been used on these horses and trimmed the hooves so that they were balanced. Horses received the same basic trim regardless of which group they would be in. None of the horses had a history of any serious lameness (including laminitis or navicular), they remained free of lameness throughout the study, and none of the horses had obviously hoof abnormalities (no concave dorsal hoof walls, etc.) The horses did have a varied exercise history, with some horses having competed extensively and others having no performance record. However, all the horses had been retired for at least 12 months prior to the beginning of the study. All procedures were approved by the University of Melbourne Animal Ethics Committee (1011571.1).

### 2.1. Statistical Analysis

An analysis of variance (ANOVA) appropriate to a crossover design was used to assess the effect of treatment on the change in hoof shape over a seven-week period. The model included the effects of treatment (shod and barefoot), sequence (error term animal within sequence), and period (one and two). Stata (Version 12.1, StataCorp, College Station, TX, USA) was used for the analysis. Statistical significance was set at *p* < 0.05. The left and right front hooves were analysed separately for ease of interpretation.

### 2.2. Repeatability

Repeatability was previously assessed in a separate study by measuring fifty horses (both forelimbs) two times each. The repeatability coefficient describes the range within which 95% of absolute differences between two readings from the same horse would be expected to lie [16]. The repeatability coefficient was defined as 1.96(√2)Sw, where Sw is the square root of the residual mean square from a one-way analysis of variance with horse as the factor. WinPepi (Version 11.24, J. H. Abramson, Hebrew University, Jerusalem, Israel) was used for the statistical analysis of repeatability. 

## 3. Results

The results are displayed in Table 1, Table 2, Table 3 and Table 4. Results are displayed as the mean difference in values for the horses when shod versus barefoot ± the SEM for the left (L) and right (R) front hooves.

### 3.1. Proximal Hoof Circumference

PHC decreased during both seven-week periods under both treatments. This decrease was significantly greater (L *p* = 0.0026; R *p* = 0.0002) when horses were shod, compared to barefoot. 

### 3.2. Hoof Angle

Hoof angle increased slightly (became steeper) when horses were barefoot but decreased (became less steep) when they were wearing shoes. The difference between treatments (shod and barefoot) was significant (L *p* = 0.0004; R *p* = 0.0079).

### 3.3. Heel Width and Heel Height

Heel width, lateral heel height, and medial heel height all showed an average increase in both hooves under both treatments, however none of the differences between treatments were significant (all *p*-values > 0.05).

### 3.4. Solar Measurements

Sole length decreased in both treatments over the seven-week period. Sole length decreased more when horses were barefoot. This difference was significant only for the right fore (L *p* = 0.4819; R *p* = 0.0010). Sole width increased in both treatments over the seven-week period. This increase was not different between treatments (*p* > 0.05). 

Solar circumference increased when horses were barefoot and decreased when horses were shod; this change was significant (L *p* = 0.0182; R *p* = 0.0010). Solar area increased more when horses were barefoot on average, but this change was not significant (*p* > 0.05).

### 3.5. Hoof Growth and Hoof Wear 

Horses showed an increase in dorsal hoof growth cross mark for the right and left hooves, and a decrease in the dorsal hoof wear cross mark, under both treatments. There was a significant difference between treatments for dorsal hoof wear (L *p* = 0.0005; R *p* = 0.0169), but not for dorsal hoof growth (*p* > 0.05).

Both treatment groups showed an increase in lateral hoof growth cross mark and a decrease in the lateral hoof wear cross mark, for the left and right hooves with and without shoes. The differences between shod and barefoot were nonsignificant (all *p*-values > 0.05). 

Both treatment groups had an increase in medial hoof growth cross mark for both front hooves and a decrease in medial hoof wear cross mark. Neither variable exhibited a significant difference between treatments (all *p*-values > 0.05). 

### 3.6. Position of the Frog

The distance from the frog to the wall of the hoof increased in all directions in both treatments. All increases were greater when the horses were barefoot, except for the distance from frog to toe on the right front hoof, which was greater when the horses were shod. Changes in the distance from the frog to the lateral wall were nonsignificant between treatments (*p* > 0.05). Changes in the distance from the frog to the medial wall were significant between treatments for the left hoof (L *p* = 0.0466), but not for the right hoof (R *p* = 0.5016). Changes in the distance from the frog to the toe were nonsignificant between treatments (*p* > 0.05).

Horses showed changes in body weight during both seven-week treatment periods. The results are reported as the mean ± the standard deviation. During the first data collection the mean body weight was 417 ± 36 kg (range 340–455 kg). At the mid-point the mean body weight was 412 ± 33 kg (range 343–448 kg). At the final data collection, the mean body weight was 419 ± 28 kg (range 357–445). The mean change was −4.9 kg in period one (*p* = 0.18) and 15.2 kg (*p* = 0.80) in period two. When the change in body weight was analysed by treatment the horses gained 1.4 kg more when they were barefoot compared to shod, but this difference was nonsignificant (*p* = 0.84).

### 3.7. Body Weight

Horses showed changes in body weight during both seven-week treatment periods. The results are reported as the mean ± the standard deviation of the mean. During the first data collection the mean body weight was 417 ± 36 kg (range 340–455 kg). At the mid-point the mean body weight was 412 ± 33 kg (range 343–448 kg). At the final data collection, the mean body weight was 419 ± 28 kg (range 357–445). The mean change was −4.9 kg in period one (*p* = 0.18) and 15.2 kg (*p* = 0.80) in period two. When the change in body weight was analysed by treatment the horses gained 1.4 kg more when they were barefoot compared to shod, but this difference was nonsignificant (*p* = 0.84).

### 3.8. Repeatability

The repeatability of the measurements used in this study are displayed in Appendix A. 

## 4. Discussion

To the best of the authors’ knowledge, this study is the first of its kind to demonstrate differences in hoof shape when horses were shod versus barefoot. The results show significant differences in PHC, hoof angle, wear at the toe (dorsal cross mark two), sole length, and solar circumference. 

### 4.1. Proximal Hoof Circumference

All horses involved in the study showed a decrease in PHC over the course of fourteen weeks. This result could be related to the season [12], substrate hardness [17], or a combination of both. Interestingly, horses had a significantly larger decrease in PHC when shod compared to barefoot. It could be possible that proprioceptive feedback from the hoof striking the ground influences PHC. A lack of input from the hoof wall contacting the ground (due to the shoe) may cause a more rapid decrease in PHC. In addition, the steel shoe may limit the deformation of the hoof upon impact, hence influencing PHC. Roepstorff et al. noted that the application of a horseshoe raised the hoof from the ground by supporting the hoof wall, and resulted in less expansion of the palmar aspect of the hoof wall when compared with unshod horses [18]. Another possibility is that shoeing causes slight but significant differences in the movement and loading of the distal limb even when the horse is accustomed to wearing standard iron shoes [9], hence changes in movement of the shod horses may have contributed to differences in PHC between treatments. Hagen and colleagues found that slight differences in shoe type could impact limb biomechanics [19]. A final consideration is that the additional weight of the horseshoe, which is lifted each time the horse picks up its hoof, may cause a change in the muscle tone or fascia of the leg and corresponding changes in PHC. Research has shown a decrease in the PHC of racehorses, which is reversed if the horse is turned out with the shoes removed [11], therefore, this study supports the idea that the application of a shoe could be partially responsible for this change.

### 4.2. Hoof Angle

Hoof angle increased when horses were barefoot, becoming slightly and significantly steeper, and decreased, developing a significantly more acute angle, when they were wearing shoes. These data suggest that shod and unshod horses exhibit different changes in hoof angle during a seven-week shoeing interval. This difference is likely related to the increased wear of the unshod hoof, allowing it to maintain a steeper hoof angle. Previous research suggests that changes in hoof angle between shoeing sessions were associated with an increase in dorsal length of the hoof [20]. Moleman et al. also described a decrease in hoof angle (measured from radiographs) from 55.4 ± 3.2° to 51.9 ± 3° (mean ± SEM) over an eight-week shoeing interval in nine horses [21]. Additionally, the study by van Heel and colleagues demonstrated a significant shift in the location of the center of pressure under the hoof and an increased moment around the distal interphalangeal joint [21], demonstrating the influence of changes in hoof angle on the internal structures of the foot. Researchers have highlighted the importance of hoof angle to joint health, noting that hoof angle has a direct effect on the pastern and coffin joints and inversely effects the fetlock [22]. Clayton found that acute hoof angulation, an increase of 9–11°, significantly increased breakover time in both the front and hind limbs [23]. Horses in the Clayton study were also more likely to strike the ground with toe first impact with an acute angulation; toe first impact is generally only seen in lame horses [23,24]. The changes observed during this study, a change of approximately −1.2° in shod horses, were much smaller in magnitude but still show the natural changes in hoof angle during a shoeing interval. Keeping the horse in shoes for a longer period of time before resetting could contribute to larger decreases in hoof angle and this factor should be considered when designing shoeing intervals.

### 4.3. Heel Width and Heel Height

There were no significant differences between treatments for heel width or heel height. Heel width increased in both treatments over the course of the study. Heel width increased more when the horses were barefoot, but the difference was nonsignificant. Future research could explore the relationship between horseshoes and heel expansion, especially when the shoe is worn for extended periods of time.

Both lateral and medial heel height increased in both treatments, but the difference was not significant. It could be expected that heel height would increase in shod horses (with the shoe protecting the heel area from wear) and decrease in barefoot horses. It should be noted that heel height is difficult to measure with a high repeatability value using the methods in this study (see Section 4.8).

### 4.4. Solar Measurements

Sole length decreased in both treatments over the seven-week period. The decrease was greater when the animal was barefoot, but this was only significant for the right fore. This is an interesting finding, because we would expect the sole length to increase as the hoof grew, but the decrease could be related to hoof wear at the toe. There was a slightly larger decrease in sole length when the horses were barefoot. 

Sole width increased in both treatments over the seven-week period. Hoof wear increased more when the horse was barefoot, but this difference was nonsignificant. This is most likely due to the increased flaring at the distal wall of the hoof in barefoot horses. The shoe could limit this deformation in the shod horse.

Solar circumference was significantly different between treatments for both hooves. There was a small increase in solar circumference when the horses were barefoot, consistent with the spreading of the hoof. When horses were shod there was a small decrease in hoof circumference, indicating that the shoe may have restricted hoof spreading on the solar surface. Solar area increased when horses were barefoot. When horses were shod, the solar area increased only slightly for the left fore and decreased slightly for the right fore. The changes in solar area were not significantly different between treatments.

### 4.5. Hoof Growth and Hoof Wear

There were no significant differences in hoof growth when the horses were shod versus barefoot. Only a few other studies have focused on the difference between hoof growth and hoof wear, by marking the horse hoof and measuring hoof growth from the coronet band to the mark and hoof wear from the mark to the ground [12,25]. 

The mean growth at the dorsal growth cross mark for barefoot horses was 0.238 mm per day for the left fore and 0.235 mm per day for the right fore. Florence and McDonnell reported a growth of 0.33–0.35 mm ± 0.02 (mean daily growth ± SE) at the dorsal midline of the hoof. They collected data from forty barefoot self-trimming ponies measured from July to September [12]. Reilly et al. reported hoof growth in twelve stabled control horses of 0.18 mm ± 0.09 mm (daily average growth ± SD) from October to April [25]. Altogether, these data present a range that includes those values presented in our study. Additional factors could have influenced hoof growth including exercise rate, nutrition, loading patterns, etc. These horses were kept in similar conditions, turned out together and fed the same diet to try to minimise the variables.

Vermunt and Greenough suggested that hoof trimming may stimulate hoof growth in cattle [26]. The horses studied were maintained on a seven-week trimming schedule, so trimming should not have acted as a stimulant for growth. However, future studies could collect weekly growth data to investigate this effect. Some anecdotal evidence has suggested that the hoof grows better unshod. At all locations around the hoof, the hoof grew slightly less when the horse was wearing shoes, but this was nonsignificant. These horses were only maintained with shoes on for seven weeks and additional research could focus on hoof growth in horses maintained in shoes for longer. The season has also been associated with a changed growth rate in free-ranging ponies [12]. The current study measured horses during a summer to fall period, thus season could have affected the hoof by continually decreasing growth across the experimental period. However, there were no significant differences in growth between period 1 and period 2 (data available but not shown). 

Unshod subjects had a mean wear of −0.17 mm for the left fore and −0.18 mm for the right fore. Florence and McDonnell reported slightly greater wear, with a mean of 0.24 ± 0.03 mm to 0.52 ± 0.04 mm [12]. Our subjects exhibited wear at all three locations around the hoof (dorsal, medial, and lateral) in both treatments, despite the protection provided by the shoe. It is possible that the hoof still contacts the ground (especially if the horses are sinking into soft ground), or there is friction between the hoof wall and the shoe. Barrey’s research showed a greater loading of the caudal area of the hoof upon landing [27], although Barrey’s study did not directly measure wear. Wear at all hoof locations even with a horseshoe applied could be related to the hydration of the hoof. It could also be related to collapse of the hoof horn tubules [14]. The hoof horn tubules could collapse at the distal border of the hoof making it appear that the hoof was more worn. This explanation would also account for the decrease in sole length over time as the heel collapsed under the foot and the sole migrated forward.

For this study, more wear was predicted when the hoof was not protected by a shoe. However, our results show no significant difference in wear in the unshod versus shod treatments, except at the toe. The toe was the area with the greatest amount of wear in unshod horses, and the significant difference in wear between treatments at this location demonstrates the protective nature of the shoe.

### 4.6. Position of the Frog

The distance from the frog to the hoof wall increased in all three directions (medial, lateral, toe), indicating that the solar area of the hoof was growing or spreading. There were no significant differences between shod and barefoot horses except the distance from the frog to the medial hoof wall showed significantly less spreading in the shod horses. The fact that only the left hooves showed a significant difference could be due to numerous factors, including limb preference or handedness (horses were not assessed for limb preference in this study). Several studies have identified asymmetries between the left and right limbs. Some level of asymmetry is likely to be biologically normal and associated with horse-level laterality [28]. 

The lack of significant differences between treatments for many of the solar values could be due to the fact that horses had wear of the distal edges of the hoof wall in both treatments. There was a significant difference between treatments for dorsal wear, therefore it would be expected that the frog to toe variables would also differ between treatments. There was no significant difference, perhaps because the heel became more underrun as the toe grew out, and hence the position of the frog in relation to the toe did not change. It is also possible that changes to the frog and sole happened simultaneously, and thus changes in the position of the frog were not detected or that a small change was undetected by our methods.

### 4.7. Body Weight

During the first seven-week period the horses had a mean decrease in body weight and in the second seven-week period they showed a mean increase in body weight. When the change in body weight was analysed by treatment, the horses gained 1.4 kg more when they were barefoot compared to shod but this difference was nonsignificant (*p* = 0.84). The horses were measured with a commercially available body weight tape and this is a limitation of the study. Several studies have evaluated the accuracy of different commercially available weight tapes and found that there is a large variability in weight estimated and low correlation with body weight measured on a scale [29]. To mitigate this effect, each weight measurement used is the average of two separate measurements and the same weight tape was used throughout the study by the same investigator.

### 4.8. Repeatability

The repeatability of the measurements used in this study are displayed in Appendix A. The smaller the repeatability coefficient the more agreement between measurements, indicating a higher repeatability of that measurement method [16]. A measurement may be said to be repeatable when the repeatability coefficient is smaller than a pre-determined criterion. For the purposes of this study the repeatability should be smaller than the difference seen over the course of the seven weeks. PHC has a repeatability coefficient for this operator of 0.1 cm, indicating that multiple measurements agree within 1 mm. The difference in PHC across this study ranged from 0.5 to 1 cm, so the authors feel confident that they were able to detect changes in PHC. Other measurements were more difficult. For example, the repeatability coefficient of heel height was around 5 mm. The changes in heel height during this study ranged from 3 to 7 mm, making the authors less confident in their ability to capture changes in variables such as heel height.

### 4.9. Limitations

One horse was dropped from the study midway through for reasons unrelated to the data collection, which made the group sizes uneven with five horses shod in period one and six in period two. This unbalanced design was considered in the statistical analyses. The number of horses (n = 11) used was also relatively small. We were able to distinguish statistical differences with this small group, but more horses would have been advantageous. Measurements that were taken while the foot was loaded, such as PHC, may have been influenced by changes in body weight. There were no significant differences in body weight from period 1 to period 2, but this should still be considered. A separate study done by this lab indicated little, to no differences in PHC when horses gained a considerable amount of weight over a 60-day period, however that data has not been published.

## 5. Conclusions

This study demonstrates a significant effect of shoeing on hoof shape. Horses that were shod displayed a larger decrease in proximal hoof circumference, a decrease in hoof angle, a reduction in solar circumference, and less wear at the toe than occurred when they were barefoot. This study is the first to describe morphometric changes in hoof shape between horses when they are shod versus barefoot. It is the first study to demonstrate a change in PHC related to shoeing. A better understanding of the factors that influence hoof shape may lead to better hoof management practices that minimise the risk of injury to the horse.

## Figures and Tables

**Table 1:** The starting values at the beginning of the study displayed as the mean (*n* = 11), standard deviation (SD), and range for the left and right front hooves.

Variable	Left			Right		
	Mean	SD	Range	Mean	SD	Range
Age (years)	13	5.9	6–22			
Weight (kgs)	416.9	36.3	340–455			
PHC (cm)	32.9	1.1	30.7–34.8	33.1	1.0	31.7–34.7
Hoof Angle (°)	47.9	1.8	45.0–50.0	48.2	1.9	45.0–51.0
Sole Width (mm)	112.0	6.3	99.0–122.0	111.3	7.1	96.5–124.5
Heel Width (mm)	56.0	6.0	48.0–67.5	54.6	7.3	42.0–69.0
Sole Length (mm)	123.0	4.7	115.5–130.0	123.6	3.8	118.5–130.0
Lateral Heel Height (mm)	24.2	2.8	20.0–29.5	26.3	4.6	20.0–35.5
Medial Heel Height (mm)	23.2	2.8	17.0–27.0	25.0	2.9	21.5–30.0
Solar Area (cm^2^)	133.2	10.0	115.1–153.3	130.7	10.0	116.0–150.3
Solar Circum. (cm)	44.5	1.9	41.1–48.0	44.3	1.6	41.5–47.2

**Table 2:** Changes in hoof shape parameters when horses were barefoot versus shod over a seven-week period. The results are displayed as the mean change when horses were barefoot (*n* = 11) versus shod (*n* = 11), followed by the difference between the two treatments and the standard error of the mean (SEM) difference. Results are displayed for the left (L) and right (R) forelimbs.

Variable	Barefoot Change	Shod Change	Shod-Barefoot	SEM	*p*-Value
Proximal Hoof Circumference L (cm)	−0.65	−1.30	−0.65	0.16	0.0026
Proximal Hoof Circumference R (cm)	−0.66	−1.44	−0.78	0.13	0.0002
Hoof Angle L (°)	0.49	−1.21	−1.70	0.31	0.0004
Hoof Angle R (°)	0.63	−1.22	−1.84	0.54	0.0079
Heel Width L (mm)	7.01	4.17	−2.83	2.34	0.2561
Heel Width R (mm)	6.20	2.43	−3.77	2.04	0.0981
Lateral Heel Height L (mm)	5.80	5.88	0.08	2.07	0.9719
Lateral Heel Height R (mm)	6.03	4.83	−1.20	0.69	0.1142
Medial Heel Height L (mm)	3.66	3.02	−0.63	1.13	0.5883
Medial Heel Height R (mm)	7.00	6.05	−0.95	1.91	0.6311
Sole Length L (mm)	−3.14	−2.18	0.97	1.32	0.4819
Sole Length R (mm)	−8.40	−3.33	5.07	1.06	0.0010
Sole Width L (mm)	7.98	4.84	−3.13	1.54	0.0720
Sole Width R (mm)	7.47	4.72	−2.75	1.44	0.0881
Solar Circumference L (cm)	1.10	−0.09	−1.19	0.41	0.0182
Solar Circumference R (cm)	1.26	−0.25	−1.50	0.31	0.0010
Solar Area L (cm^2^)	3.82	0.63	−3.19	1.79	0.1093
Solar Area R (cm^2^)	1.68	−0.65	−2.34	1.64	0.1875

**Table 3:** Changes in hoof growth and wear of horses when barefoot and shod during a seven-week period. The results are displayed as the mean change when horses were barefoot (*n* = 11) and shod (*n* = 11), followed by the difference between the two treatments and the standard error of the mean (SEM) difference. Results are displayed for the left (L) and right (R) forelimbs.

Growth and Wear (mm)	Barefoot Change	Shod Change	Shod-Barefoot	SEM	*p*-Value
Dorsal Growth L	11.66	9.66	−2.00	1.28	0.1539
Dorsal Growth R	11.53	9.38	−2.16	1.06	0.0728
Dorsal Wear L	−8.30	−2.88	5.43	1.01	0.0005
Dorsal Wear R	-8.56	−3.71	4.85	1.66	0.0169
Lateral Growth L	9.71	8.15	−1.56	1.49	0.3224
Lateral Growth R	10.05	9.63	−0.43	0.80	0.6088
Lateral Wear L	−5.77	−4.46	1.31	1.53	0.4148
Lateral Wear R	−1.76	−3.15	−1.38	0.87	0.1456
Medial Growth L	10.81	9.14	−1.67	1.56	0.3135
Medial Growth R	9.90	9.00	−0.90	1.09	0.4316
Medial Wear L	−2.43	−1.39	1.04	1.03	0.3384
Medial Wear R	−6.14	−6.13	0.01	2.01	0.9968

**Table 4:** Changes in the position of the frog in relation to the sole of the hoof during seven-weeks in horses when they were shod and barefoot. The results are displayed as the mean change when horses were barefoot (*n* = 11) and shod (*n* = 11), followed by the difference between the two treatments and the standard error of the mean (SEM) difference. Results are displayed for the left (L) and right (R) forelimbs.

Frog Variables (mm)	Barefoot Change	Shod Change	Shod-Barefoot	SEM	*p*-Value
Frog to Lateral Wall L	3.82	2.81	−1.01	1.32	0.4649
Frog to Lateral Wall R	5.01	2.40	−2.61	1.56	0.1296
Frog to Medial Wall L	4.13	1.28	−2.86	1.24	0.0466
Frog to Medial Wall R	2.27	0.96	−1.31	1.87	0.5016
Frog to Toe L	3.85	1.69	−2.16	1.70	0.2354
Frog to Toe R	2.79	3.53	0.74	1.53	0.6391

## Appendix A

**Table A1:** Repeatability of the measurements for the left and right front hooves (*n* = 50). Results are displayed as the repeatability coefficient (RC) followed by the 95% confidence interval (CI).

Variable	Left RC	95% CI	Right RC	95% CI
Proximal Hoof Circumference (cm)	0.1	0.1–0.1	0.1	0.1–0.2
Hoof Angle (°)	1.1	0.9–1.3	0.9	0.8–1.1
Hoof Growth Mark (mm)	2.5	2.0–3.0	2.7	2.3–3.4
Hoof Wear Mark (mm)	2.3	2.0–2.9	2.0	1.7–2.5
Frog to Lateral Wall (mm)	2.5	2.1–3.1	2.2	1.8–2.7
Frog to Medial Wall (mm)	1.8	1.5–2.2	1.9	1.6–2.3
Frog to Toe (mm)	1.9	1.6–2.4	1.8	1.5–2.3
Heel Width (mm)	3.2	2.7–4.0	3.1	2.6–3.9
Lateral Heel Height (mm)	5.2	4.3–6.5	3.7	3.1–4.6
Medial Heel Height (mm)	5.0	4.2–6.3	5.3	4.4–6.6
Sole Width (mm)	2.1	1.7–2.6	2.0	1.7–2.5
Sole Length (mm)	5.0	4.2–6.2	4.4	3.7–5.5
Solar Circumference (cm)	2.1	1.8–2.7	2.3	2.0–2.9
Solar Area (cm^2^)	10.2	8.5–12.7	10.1	8.4–12.5
